# No Association between Circulating Levels and Genetic Variants of IL-6 and TNF-α and Colon Adenoma

**DOI:** 10.4021/gr529w

**Published:** 2013-05-03

**Authors:** Caila B. Vaughn, Heather M. Ochs-Balcom, Jing Nie, Zhengyi Chen, Cheryl L. Thompson, Russell Tracy, Li Li

**Affiliations:** a Department of Social and Preventive Medicine, University at Buffalo, Buffalo, NY, United States; b Department of Family Medicine and Community Health, Case Western Reserve University, Cleveland, Ohio, United States; c Department of Biochemistry, University of Vermont, Burlington, Vermont, United States

**Keywords:** Interleukin-6, Colon adenoma, Tumor necrosis factor alpha, Single nucleotide polymorphisms

## Abstract

**Background:**

Interleukin 6 (IL-6) and tumor necrosis factor alpha (TNF-α), two important inflammatory cytokines, have been inconsistently associated with risk of colon neoplasia in epidemiological studies. However, research to date has not adequately assessed whether race-specific differences may exist in associations between biomarkers and genetic variants of these cytokines and colorectal adenoma - the precursor lesions of colorectal cancer. We sought to determine whether circulating levels of IL-6 and TNF-α, or genetic polymorphisms in IL-6and TNF*-*α were associated with colon adenoma and if so, whether that association differed by race.

**Methods:**

We analyzed the associations of circulating levels and single nucleotide polymorphisms (SNPs) of IL-6 and TNF-α with risk of colon adenomas in a colonoscopy -based case-control study of 401 incident adenoma cases and 1,050 controls. We used multivariate unconditional logistic regression models to estimate the odds ratios (OR) and 95% confidence intervals (95% CI) for levels or genotypes (log additive models) of IL-6 and TNF-α.

**Results:**

Compared to the bottom tertile of IL-6, the adjusted ORs were 1.06 (0.75 - 1.44) and 1.01 (0.72 - 1.40), respectively for the 2nd and 3rd tertiles (P_trend_ = 0.10); the corresponding ORs for TNF-α were: 0.85 (0.63 - 1.15) and 1.01 (0.75 - 1.36), respectively (P_trend_ = 0.39). Race-stratified analyses did not reveal any significant associations. There were also no statistically significant associations between IL-6 and TNF-α SNPs and colon adenoma.

**Conclusions:**

Our results do not support pre-diagnostic levels of IL-6, TNF-α or their genetic variants as significant risk factors for the development of colon adenoma.

## Introduction

Chronic inflammation is widely recognized as an important pathway for the development of colon neoplasia [1]. Interleukin-6 (IL-6) and tumor necrosis factor-α (TNF-α) are two important inflammatory cytokines that have been implicated in human colon carcinogenesis [2-9]. Recent evidence indicates that serum levels of IL-6 and TNF-α may be elevated in those with colorectal adenoma [4, 6]. However, epidemiological studies of the associations of IL-6 and TNF-α with risks of colorectal cancer or adenoma, precursor lesions of cancer, however, have been inconsistent [1, 4-6]. Because inflammation is now widely considered to be a key component of carcinogenesis, it is important to attempt to rectify these inconsistencies in the epidemiologic literature. It is also important to elucidate whether circulating levels of these cytokines impacts the initiation and development of colorectal carcinogenesis, as would be expected if they are associated with adenoma, or whether these cytokines impact the progression of colorectal carcinogenesis.

Also, significant racial disparities exist in colon carcinogenesis. African Americans have higher incidence and mortality from colorectal cancer than do Caucasians [1, 10-12]. However, studies to date have not adequately assessed whether these associations may vary by race or be potentially modified by race. Because such disparities exist, it is important to determine whether the mechanisms at play for carcinogenesis in Caucasians are similar to those in African Americans. Specifically in this study we sought to determine whether inflammation, as measured by inflammatory cytokines IL-6 and TNF-α, exhibited similar associations with colon adenomas in both Caucasians and African Americans in our study.

We investigated the associations between circulating levels and genetic variants of IL-6 and TNF-α and colon adenomas in a case-control study including 894 Caucasians and 557 African Americans who underwent routine colonoscopy at University Hospitals Case Medical Center, Cleveland, Ohio. We also evaluated the association of SNPs in the IL6 and TNF-α genes and colon adenomas.

## Materials and Methods

Participants were recruited for the Case Transdisciplinary Research on Energetics and Cancer (TREC) Colon Polyps Study [13]. In brief, patients without personal history of colorectal cancer or adenomas and undergoing routine colonoscopy were recruited. Each patient completed a computer-assisted personal interview for lifestyle and behavioral risk factor information and provided a fasting blood sample at colonoscopy. Patients were excluded if they were ever diagnosed with inflammatory bowel disease, cancer, or colon adenomas, or were younger than 30 years of age [13]. The presence or absence of adenomas was histologically determined following colonoscopy. For this analysis, we included 401 incident adenoma cases and 1,050 controls; we excluded participants if they were missing data for the relevant biomarkers in our analysis. We also included only Caucasians and African Americans in this analysis due to low numbers in other racial and ethnic groups. The UHCMC approved this study and all patients provided written informed consent [13].

Serum IL-6 was quantified using ELISAs from R&D Systems, Inc. (Minneapolis, MN) with an inter-assay CV of 3.63-6.31%. TNF-α was quantified using the Panel B method from Millipore Inc. (Billerica, MA) with an inter-assay CV of 5.61-10.44%.

We selected haplotype-tagging SNPs using the Genome Variation Server (GVS) (http://gvs.gv.washington.edu/GVS/) from within IL6 and TNF-α genes as well as 5kb up- and downstream. Tag SNPs were identified using GVS from the HapMap Yoruba population with an r^2^ threshold of 0.8, 85% data coverage and 70% clustering. We restricted to SNPs with a minor allele frequency ≥ 5%. We genotyped 4 SNPs in the IL6 gene and 2 SNPs in the TNF-α gene using a Custom GoldenGate Panel (Illumina, Inc., San Diego, CA).

We examined the association of circulating IL-6 and TNF-α and colon adenomas in multivariate logistic regression models with adjustment for age, sex, smoking status, non-steroidal anti-inflammatory (NSAID) use, body mass index (BMI) and first degree family history of colorectal cancer. We categorized individuals as never smokers if they smoked less than 6 months in his or her lifetime. For NSAID use, we classified an individual as a “user” if he or she reported having taken aspirin or ibuprofen at least twice per week for at least one month. BMI was calculated as kilograms divided by height in meters squared (kg/m^2^). We also estimated IL6 and TNF-α SNP-adenoma associations using log additive genetic models with adjustment for covariates. The excellent representation of African Americans (38%) in our study further allowed for stratified analyses to assess potential racial differences in the risk associations.

## Results

Table 1 depicts the descriptive characteristics of our study sample. There were no statistically significant differences in circulating levels of IL-6 and TNF-α by case status for Caucasians or African Americans. In both Caucasians and African Americans, cases were older and more likely to be male than the controls. For the adiposity indicators that we measured, there were differences by race and case status. In Caucasians, cases had higher BMI, waist circumference and waist-to-hip ratio than controls (all P < 0.005). However, in African Americans, cases and controls did not significantly differ by BMI or waist circumference; the only difference was that cases had significantly higher waist-to-hip ratio than controls (P = 0.001).

**Table 1:** Characteristics of Study Sample by Race and Case/Control Status

Characteristic*	Caucasians			African Americans		
	Cases (n = 227)	Controls (n = 667)	P-value	Cases (n = 174)	Controls (n = 383)	P-value
Age, yr	57.1 (8.0)	54.5 (8.7)	0.001	58.1 (8.3)	54.8 (8.6)	0.001
Sex, % male	51.5	38.4	0.001	40.2	24.8	0.001
Body mass index, kg/m^2^	29.0 (6.2)	27.7 (5.9)	0.005	31.6 (7.9)	32.1 (7.7)	0.45
Waist circumference, cm	39.0 (6.6)	37.1 (6.3)	0.001	41.4 (7.9)	40.8 (7.0)	0.37
Waist-to-hip ratio	0.92 (0.09)	0.90 (0.09)	0.001	0.96 (0.09)	0.93 (0.09)	0.001
NSAID use	41.0	34.5	0.08	36.2	35.5	0.87
Smoking status:			0.37			0.02
Never	46.3	51.1		30.5	38.9	
Former	41.4	39.0		32.8	35.5	
Current	12.3	9.9		36.8	25.6	
Pack years of smoking	12.2 (26.2)	14.5 (107.3)	0.75	15.1 (20.6)	9.9 (23.7)	0.01
Family history of colorectal cancer, %	28.2	25.2	0.37	16.1	23.8	0.04
IL-6, pg/mL	2.5 (2.1)	2.2 (1.8)	0.07	3.7 (2.7)	3.4 (2.3)	0.10
TNF-α, pg/mL	5.1 (11.3)	4.2 (2.5)	0.23	4.5 (2.7)	4.6 (6.0)	0.78
Insulin, µIU/mL	7.5 (11.5)* *(n = 182*)*	6.0 (7.1)* *(n = 556*)*	0.10	15.3 (61.5) (n = 129)	10.0 (14.2) (n = 261)	0.19
Fasting glucose, mg/dL	86.3 (17.3)* *(n = 184*)*	85.3 (24.6)* *(n = 569*)*	0.59	97.9 (29.1) (n = 129)	94.2 (37.5) (n = 262)	0.33

* Values for continuous variables are mean (SD); for categorical variables, values are for percentages. Two-sided comparisons were computed using independent samples t-tests for continuous and by χ square test for categorical variables.

In Caucasians, cases and controls had mean IL-6 levels of 2.5 pg/mL and 2.2 pg/mL, respectively (P = 0.07). In African Americans, cases and controls had mean IL-6 levels of 3.7 pg/mL and 3.4 pg/mL, respectively, (P = 0.10). For TNF-α, Caucasian cases and controls had mean levels of 5.1 pg/mL and 4.2 pg/mL, respectively (P = 0.23). In African Americans, cases and controls had mean TNF-α levels of 4.5 pg/mL and 4.6 pg/mL (P = 0.78).

In the entire study population, IL-6 level is positively associated with risk of colon adenomas (P-trend = 0.002) in crude analysis; however this association is reduced to non-significance with adjustment for covariates (P-trend = 0.10) (Table 2). Race-stratified analyses revealed no significant association for IL-6 among Caucasians or African Americans. There is no association between circulating levels of TNF-α and colon adenomas in either the crude or adjusted models (adjusted P-trend = 0.53 in Caucasians and P = 0.13 in African Americans).

**Table 2:** Association of Colon Adenomas and Circulating IL-6 and TNF-α

Category^*^	Caucasian				African-American				All subjects			
	Cases n = 227	Controls n = 667	Crude OR (95% CI)	Adjusted OR^**^ (95% CI)	Cases n = 174	Controls n = 383	Crude OR (95% CI)	Adjusted OR^**^ (95% CI)	Cases n = 401	Controls n = 1,050	Crude OR (95% CI)	Adjusted OR^***^ (95% CI)
IL-6												
Tertile 1	73	224	1.00	1.00	62	129	1.00	1.00	113	352	1.00	1.00
Tertile 2	67	221	0.93 (0.64 - 1.36)	0.77 (0.52 - 1.16)	43	128	0.70 (0.44 - 1.11)	0.58 (0.36 - 0.95)	136	348	1.22 (0.91 - 1.63)	1.06 (0.78 - 1.44)
Tertile 3	87	222	1.20 (0.84 - 1.73)	0.87 (0.57 - 1.32)	69	126	1.14 (0.75 - 1.74)	1.00 (0.62 - 1.61)	152	350	1.35 (1.02 - 1.80)	1.01 (0.72 - 1.40)
p for trend			0.05	0.53			0.08	0.13			0.002	0.10
TNF-α												
Tertile 1	71	222	1.00	1.00	51	128	1.00	1.00	123	350	1.00	1.00
Tertile 2	66	223	0.93 (0.63 - 1.36)	0.78 (0.52 - 1.16)	54	128	1.06 (0.67 - 1.67)	1.06 (0.66 - 1.71)	120	351	0.97 (0.73 - 1.30)	0.85 (0.63 - 1.15)
Tertile 3	90	222	1.27 (0.88 - 1.82)	0.94 (0.63 - 1.39)	69	127	1.36 (0.88 - 2.11)	1.14 (0.72 - 1.81)	158	349	1.29 (0.98 - 1.70)	1.01 (0.75 - 1.36)
P for trend			0.23	0.20			0.78	0.55			0.20	0.39

^*^For Caucasians, the IL-6 cutoffs were 1.25 pg/mL& 2.22 pg/mL; for African American, 2.00 pg/mL & 3.83 pg/mL; for all subjects, cutoffs were 1.44 pg/mL & 2.71 pg/mL. For Caucasian, the TNF-α cutoffs were 3.02 pg/mL & 4.50 pg/mL; for African Americans, 3.00 pg/mL & 4.42 pg/mL; for all subjects, cutoffs were 3.01 pg/mL &4.46 pg/mL.**Adjusted for age, sex, non-steroidal anti-inflammatory use, BMI, family history of colorectal cancer, and smoking status. ***Also adjusted for race.

Table 3 shows the genetic association results for IL6 and TNF-α. We found no statistically significant association between any SNPs and colon adenomas in crude (data not shown) or adjusted models.

**Table 3:** Association of IL-6 and TNF-α Genotypes With Colorectal Adenomas

Gene	SNP	Alleles (m/M)	MAF cases	MAF controls	Caucasians Adjusted OR* (95% CI)	African Americans Adjusted OR* (95% CI)	All Subjects Adjusted OR** (95% CI)
IL-6	rs2069840	G/C	0.23	0.27	0.89 (0.71 - 1.13)	0.75 (0.50 - 1.11)	0.86 (0.71 - 1.06)
IL-6	rs1554606	A/C	0.39	0.39	1.08 (0.87 - 1.34)	0.92 (0.69 - 1.23)	1.01 (0.85 - 1.21)
IL-6	rs2069827	T/G	0.05	0.06	0.86 (0.58 - 1.29)	0.82 (0.28 - 2.40)	0.84 (0.57 - 1.22)
IL-6	rs2069849	A/G	0.08	0.07	1.47 (0.78 - 2.76)	0.93 (0.64 - 1.35)	1.05 (0.76 - 1.45)
TNF-α	rs3093662	G/A	0.07	0.07	1.15 (0.78 - 1.70)	0.78 (0.45 - 1.34)	0.99 (0.72 - 1.36)
TNF-α	rs1799964	G/A	0.18	0.18	1.05 (0.80 - 1.38)	1.11 (0.77 - 1.61)	1.06 (0.85 - 1.32

* Odds ratios and 95% confidence intervals adjusted for age, sex, non-steroidal inflammatory drug use, BMI, family history of colorectal cancer, and smoking status. **In addition, adjusted for race.

## Discussion

In this colonoscopy-based study, we found no evidence for an association between pre-diagnostic circulating levels of IL-6 levels or TNF-α and colon adenomas, nor statistically significant associations with inherited genetic variation in either IL6 or TNF-α genes. While our results are null, an advantage of this study is that the relatively large proportion of African Americans in our sample allowed us to test these hypotheses in a group that has been unstudied to date. Our results may also provide information about the timing of the impact of these cytokines. It may be that these cytokines impact progression of colon carcinogenesis after adenomas have already developed.

Studies suggesting that circulating levels of IL-6 and TNF-α are associated with colorectal cancer are fairly consistent [3, 5, 7, 8]. Previous studies regarding associations between IL-6 and TNF-α levels and colorectal adenomas are inconsistent. IL-6 and TNF-α have been associated with colorectal adenoma in a few studies [4, 6] but not in others [1, 5]. Furthermore, one study that found an association between IL-6 and TNF-α was a colonoscopy-based cross-sectional study and therefore temporality of the association cannot be determined [4]. Our results suggest that these inflammatory cytokines may not be associated with the initiation of colorectal carcinogenesis in Caucasians and African Americans. Based on results from other studies which focused on the associations between these cytokines and colorectal cancer [3, 5, 7, 8], it may be that these cytokines impact the progression of colorectal cancer rather than the initiation.

In the IL6 gene, two SNPs, rs1800795 and rs1800796 have been studied in particular detail with colorectal cancer, and rs1800795 has demonstrated positive associations with colorectal cancer [14, 15]. In TNF-α, rs1800629 has been examined with colorectal cancer with inconsistent results [16]. The SNPs we genotyped in this study have been studied with regard to obesity and adiposity measures but not with colorectal cancer or adenomas. Our study was powered to detect ORs in the range of 1.23 (MAF = 0.40) to 1.38 (MAF = 0.10) in the whole sample.

Our study is not without limitations. We only measured concentration of IL-6 and TNF-α at one time point before the colonoscopy. As the adenoma was already present, this may be a case of reverse causality. However, our null findings do not support this suggestion. Still, this study is the first to our knowledge to actually examine whether these biomarker associations differed by race.

Our study is also the first to examine haplotype-tagging SNPs as well as circulating levels of IL-6 and TNF-α and risk of colon adenomas in Caucasians and African Americans separately. In summary, our results showed no evidence for association, or racial differences in the association, of IL-6 and TNF-α biomarkers or SNPs with risk of colon adenoma.
